# Supplementary material for: Interhospital variation in the nonoperative management of acute cholecystitis
Source: PLoS One. 2024 Jun 10;19(6):e0300851. doi: 10.1371/journal.pone.0300851 (PMC11164333; doi:10.1371/journal.pone.0300851)
Supplement: Attachment — Submitted filename: Response to Reviewers.docx [file pone.0300851.s001.docx]

**Response to Reviewers**

**Reviewer # 1:**

**1. the topic is not unique but worthy of researching**

**2. there are many papers in google scholar and Refseek about this topic since 2020**

**3. Ethical approval is mentioned**

**4. the title is attractive**

**5. the abstract contains plagiarism. Otherwise it is good**

Thank you for your comment. We have made the following edits within the abstract (Page 2-3).

*Cholecystectomy remains the standard management for acute cholecystitis. Given*

*that rates of nonoperative management have increased, we hypothesize the existence*

*of significant hospital-level variability in operative rates.*

*All adult admissions for acute cholecystitis were queried using the 2016-2019*

*Nationwide Readmissions Database. Centers were ranked by nonoperative rate using*

*multi-level, mixed effects modeling.*

*We noted a significant variability in the interhospital variation of the nonoperative management of acute cholecystitis.*

**6. the aim is clear**

**7. the KEYWORDS are good**

**8. lack of the abbreviations section**

**9. the introduction provide sufficient background information for readers in the immediate field to understand the problem/hypotheses**

**10. the text arrangement is good**

**11. the method section is good**

**12. the depth of the academic material is good**

**13. the study design is good**

**14. The suitability and accuracy of questions is good**

**15. The research methodology is clear**

**16. The materials are good**

**17. the logic is clear**

**18. the paper is novel**

**19. there are few grammatical errors in this article**

Thank you for your comment. We have fixed grammatical errors within the article.

**20. the related concepts are introduced**

**21. the readability is sufficient**

**22. the results are good**

**23. all figures/tables are clear enough to summarize the results for presentation to the readers**

**24. all figures/tables are well referred to in the text**

**25. the theoretical analysis in this article is sufficient**

**26. the discussion of results from multiple angles is sufficient**

**27. the conclusion is tenable**

**28. the reference section contains too many old ref**

We appreciate your comment. We have made the following amendments to the references and have minimized the usage of any older references (Pages 16-21).

*[2] Escartín A, González M, Cuello E, et al. Acute cholecystitis in very elderly*

*patients: disease management, outcomes, and risk factors for complications. Surg Res*

*Pract. 2019;2019:9709242. doi:10.1155/2019/9709242*

*[3] Parikh SS, Lindquester WS, Dhangana R. National cholecystostomy tube placement and cholecystectomy trends from 2010 to 2018. Journal of the American College of Radiology. 2023;20(6):537-539. doi:10.1016/j.jacr.2023.03.012*

*[5] Kurihara H, Binda C, Cimino MM, Manta R, Manfredi G, Anderloni A. Acute cholecystitis: Which flow-chart for the most appropriate management? Digestive and Liver Disease. 2023;55(9):1169-1177. doi:10.1016/j.dld.2023.02.005*

*[6] Kim SJ, Park HS, Lee DW. Outcome of nonoperative treatment for hip fractures in elderly patients: A systematic review of recent literature. J Orthop Surg (Hong Kong). 2020;28(2):230949902093684. doi:10.1177/2309499020936848*

*[11] Wells K, Fleshman J. Nonsurgical, minimally invasive, and surgical methods in management of acute diverticulitis. JAMA Surgery. 2019;154(2):172-173. doi:10.1001/jamasurg.2018.3112*

*[16] Using appropriate price indices for expenditure comparisons. Accessed February 15, 2024.* [*https://meps.ahrq.gov/about_meps/Price_Index.shtml*](https://meps.ahrq.gov/about_meps/Price_Index.shtml)

*[25] Mora-Guzmán I, Di Martino M, Bonito A, Jodra V, Hernández S, Martin-Perez E. Conservative management of gallstone disease in the elderly population: outcomes and recurrence. Scand J Surg. 2020;109(3):205-210. doi:10.1177/1457496919832147*

*[28] Haque LA. The effect of delays in acute medical treatment on total cost and potential ramifications due to the coronavirus pandemic. HPHR. 2021;26. Accessed February 15, 2024.* [*https://www.jstor.org/stable/48617322*](https://www.jstor.org/stable/48617322)

*[33] Popescu I, Fingar KR, Cutler E, Guo J, Jiang HJ. Comparison of 3 safety-net hospital definitions and association with hospital characteristics. JAMA Network Open. 2019;2(8):e198577. doi:10.1001/jamanetworkopen.2019.8577*

*[34] Hefner JL, Hogan TH, Opoku-Agyeman W, Menachemi N. Defining safety net hospitals in the health services research literature: a systematic review and critical appraisal. BMC Health Services Research. 2021;21(1):278. doi:10.1186/s12913-021-06292-9*

*[38] Nath JB, Costigan S, Lin F, Vittinghoff E, Hsia RY. Access to federally qualified health centers and emergency department use among uninsured and medicaid‐insured adults: california, 2005 to 2013. Mycyk MB, ed. Academic Emergency Medicine. 2019;26(2):129-139. doi:10.1111/acem.13494*

**29. please use (google scholar and Refseek) search engines then set it since 2019**

**30. the references are in order within the text**

**31. Bias is present**

**32. There is no conflict of interest with the author about this topic**

**33. Fund is mentioned**

**34. Conflict of interest is mentioned**

**35. Acknowledgement is mentioned**

**36. You can use my suggestions**

**My final decision is acceptable as it is**

Thank you for this comment. We have incorporated the above suggestions within our manuscript.

**Reviewer # 2:**

**1. Competing interests needs to be described according to the specific requirements of the journal.**

Thank you for your comment. We have provided the following statement.

*P. Benharash serves as a proctor for AtriCure. The content of this manuscript is unrelated to this work. The others authors have no disclosures. All authors declare no competing interests.*

**2. PLOS Data Policy requires authors to explain the restrictions in detail (e.g., data contain potentially identifying or sensitive patient information) and provide contact information for a data access committee, ethics committee, or other institutional body to which data requests may be sent.**

We appreciate your comment. We have added the following statement with our submission.

*The data utilized in the present study may be obtained directly from the Agency for*

*Healthcare Research and Quality (AHRQ), upon specific approval by the AHRQ for*

*data access. Users must also complete a mandatory Data Use Agreement in order to*

*access these data. For all researchers who meet these criteria, the NIS and all data of*

*the present study may be obtained directly from the AHRQ (website:* [*www.hcup-*](http://www.hcup-)

*us.ahrq.gov; email: hcup@ahrq.gov). The authors of this work had no special*

*privileges to access the data that others would not have. Legally, we are not permitted to share the datasets directly, as part of the Data Use Agreement.*

**3. Reference: The referred literature is a bit outdated so cannot reflect the latest research trends. The authors need to describe more about the new research results rather than outdated research from the past.**

Thank you for your comment. We have updated our reference list to ensure our work reflects the most current research trends. These changes can be found on (Pages 16-21).

*[2] Escartín A, González M, Cuello E, et al. Acute cholecystitis in very elderly*

*patients: disease management, outcomes, and risk factors for complications. Surg Res*

*Pract. 2019;2019:9709242. doi:10.1155/2019/9709242*

*[3] Parikh SS, Lindquester WS, Dhangana R. National cholecystostomy tube placement and cholecystectomy trends from 2010 to 2018. Journal of the American College of Radiology. 2023;20(6):537-539. doi:10.1016/j.jacr.2023.03.012*

*[5] Kurihara H, Binda C, Cimino MM, Manta R, Manfredi G, Anderloni A. Acute cholecystitis: Which flow-chart for the most appropriate management? Digestive and Liver Disease. 2023;55(9):1169-1177. doi:10.1016/j.dld.2023.02.005*

*[6] Kim SJ, Park HS, Lee DW. Outcome of nonoperative treatment for hip fractures in elderly patients: A systematic review of recent literature. J Orthop Surg (Hong Kong). 2020;28(2):230949902093684. doi:10.1177/2309499020936848*

*[11] Wells K, Fleshman J. Nonsurgical, minimally invasive, and surgical methods in management of acute diverticulitis. JAMA Surgery. 2019;154(2):172-173. doi:10.1001/jamasurg.2018.3112*

*[16] Using appropriate price indices for expenditure comparisons. Accessed February 15, 2024.* [*https://meps.ahrq.gov/about_meps/Price_Index.shtml*](https://meps.ahrq.gov/about_meps/Price_Index.shtml)

*[25] Mora-Guzmán I, Di Martino M, Bonito A, Jodra V, Hernández S, Martin-Perez E. Conservative management of gallstone disease in the elderly population: outcomes and recurrence. Scand J Surg. 2020;109(3):205-210. doi:10.1177/1457496919832147*

*[28] Haque LA. The effect of delays in acute medical treatment on total cost and potential ramifications due to the coronavirus pandemic. HPHR. 2021;26. Accessed February 15, 2024.* [*https://www.jstor.org/stable/48617322*](https://www.jstor.org/stable/48617322)

*[33] Popescu I, Fingar KR, Cutler E, Guo J, Jiang HJ. Comparison of 3 safety-net hospital definitions and association with hospital characteristics. JAMA Network Open. 2019;2(8):e198577. doi:10.1001/jamanetworkopen.2019.8577*

*[34] Hefner JL, Hogan TH, Opoku-Agyeman W, Menachemi N. Defining safety net hospitals in the health services research literature: a systematic review and critical appraisal. BMC Health Services Research. 2021;21(1):278. doi:10.1186/s12913-021-06292-9*

*[38] Nath JB, Costigan S, Lin F, Vittinghoff E, Hsia RY. Access to federally qualified health centers and emergency department use among uninsured and medicaid‐insured adults: california, 2005 to 2013. Mycyk MB, ed. Academic Emergency Medicine. 2019;26(2):129-139. doi:10.1111/acem.13494*

**4. Figures: Figures should be uploaded in TIFF or EPS format.**

We appreciate your comment. All figures have been uploaded in TIF format.

**5. Tables: Place the title above the table and the legend or footnotes below the table.**

Thank you for your comment. We have made the suggested changes in Table 1 and 2 (Pages 8-10).

**6. Page 5 line 104: Delete the comma between “including” and “age”.**

We appreciate your comment. We have adjusted this error (Page 5).

*Patient and hospital characteristics including age, sex, income quartile, primary payer, hospital setting, hospital teaching status, and bed size were defined according to the NRD data dictionary.*

**7. Methods: I would suggest adding subheadings and writing in sections to understand easier for readers.**

Thank you for your comment. We have added subheadings within the methods section to improve understanding for readers (Pages 5-7).

**8. Table 1,2：There’s no “clinical characteristics” in tables but at the title of the tables and the text.**

We appreciate your comment. We have updated the titles of the tables to better reflect the data demonstrated (Page 8 and 10).

Table 1: Baseline patient and hospital demographics of nonoperatively and operatively managed patients admitted for acute cholecystitis 2016-2019.

*Table 2: Baseline patient and hospital demographics of patients admitted for acute	cholecystitis 2016-2019 receiving treatment at Low Operative Hospitals (LOH) versus non-Low Operative Hospitals (nLOH).*

**9. Figure 2：Please explain how to definite the “hospital rank”.**

Thank you for your comment. We have included information about the hospital rank within our figure caption (Page 9).

*Figure 2: Interhospital variation in nonoperative management of acute cholecystitis*

*with 95% confidence intervals. Centers in the top decile of nonoperative rate (>9.4%) were classified as Low Operative Hospitals; LOH, Low Operative Hospitals; nLOH, non-Low Operative Hospitals.*

**10. Figure 3: The Pval for symbol “*” was not be identified.**

We appreciate your comment. We have added the symbol to Figure 3 (Page 11).
